# Emotional reactivity and its impact on neural circuitry for attention–emotion interaction in childhood and adolescence

**DOI:** 10.1016/j.dcn.2013.08.005

**Published:** 2013-09-07

**Authors:** Susan B. Perlman, Tyler C. Hein, Stephanie D. Stepp, The LAMS Consortium

**Affiliations:** University of Pittsburgh Department of Psychiatry, United States

**Keywords:** Emotion, Attention, Orbitofrontal cortex, Emotional reactivity, Development, Adolescence

## Abstract

Attention modulation when confronted with emotional stimuli is considered a critical aspect of executive function, yet rarely studied during childhood and adolescence, a developmental period marked with changes in these processes. We employed a novel, and child-friendly fMRI task that used emotional faces to investigate the neural underpinnings of the attention–emotion interaction in a child and adolescent sample (*n* = 23, age *M* = 13.46, SD = 2.86, range = 8.05–16.93 years). Results implied modulation of activation in the orbitofrontal cortex (OFC) due to emotional distractor valence, which marginally correlated with participant age. Additionally, parent-reported emotional reactivity predicted the trajectory of BOLD signal increase for fearful emotional face distractors such that participants low in emotional reactivity had a steeper latency to peak activation. Results imply that the use of the OFC to modulate attention in the face of social/emotional stimuli may mature with age and may be tightly coupled with adaptive emotional functioning. Findings are discussed in the context of risk for the development of psychiatric disorders, where increased emotional reactivity is particularly apparent.

## Highlights

• A novel, child-friendly task assessed attention–emotion neural systems.
• Activation in the OFC was modulated by valence of emotional distractor.
• Increase in activation in the OFC was found to be marginally correlated with age.
• Emotional reactivity predicted BOLD signal increase for fearful distractors.

## 1 Introduction

Executive function, described as the capacity to control and coordinate thoughts and behaviors (Elliott, 2003), is hypothesized to develop throughout late childhood and adolescence. As adolescents become increasingly independent from family members and transition into responsibility for their own daily social functioning (e.g. driving, choosing friends), adaptive executive function can aid in successful decision making rather than negative outcomes, such as school failure, drug and alcohol abuse, and juvenile delinquency (Steinberg, 2007). Executive function skills rely heavily on frontal lobe development (Goldman-Rakic, 1987, Casey et al., 1997, Rubia et al., 2003), which has been noted to peak in structural maturation during adolescence (Giedd et al., 1999). Multiple studies have also examined the development of executive functioning and its associated functional neural mechanisms during adolescence (Eigsti et al., 2006, Luna, 2009). Based upon this burgeoning literature, Steinberg (2004) has proposed an interaction between two brain networks in his research on adolescent social decision making. One of these is the cognitive-control network localized to lateral prefrontal and parietal cortex, but also includes parts of the anterior cingulate cortex. This network subserves executive functions such as planning, attention shifting, inhibitory control, and self-regulation.

Interacting with the cognitive control network in social decision making processes is the socioemotional network. This network is more sensitive to social stimuli and localized to limbic and paralimbic areas of the brain, including the amygdala and medial/orbitofrontal cortex (Steinberg, 2004). Previous studies have highlighted the interface between these brain networks by examining the impact of social/emotional information on executive functioning during adolescence. Most of this work comes from the study of inhibitory control. Hare and colleagues, for example, found that, when compared to adults and children, adolescents displayed heightened amygdala activity during a go/no-go task using emotional faces as target stimuli (Hare et al., 2008). Further, connective coupling between the amygdala and ventral prefrontal cortex was correlated with greater amygdala habituation to fearful faces. The authors describe these findings as a possible explanation for poor decision making in adolescence. Greater initial reactivity in subcortical regions, combined with immature prefrontal connectivity, considered to be inherent in the guiding of adaptive action, might contribute to inappropriate decisions in momentary emotional context.

Another executive function that becomes particularly important during this period is attention modulation, which also plays a role in effective decision making. In daily social functioning, children and adolescents must maintain attention to relevant environmental stimuli (e.g., homework) even when faced with competing emotional distractors (e.g., friends, phones, music). In one behavioral study of adolescents, scores on an emotion–attention interference task were negatively correlated with parent-reported executive function skills. The addition of executive function individual difference scores (effortful control, specifically) also predicted adolescent problem behavior, which hints at the role of attention modulation in decision making (Ellis et al., 2004). This study points to the critical role of executive functioning in the development of adolescent behavior, however, less is known about the neural mechanisms underlying attention modulation during adolescence and how it might be altered by social context. The current study focuses upon the impact of the emotion-attention interaction, both from the perspective of competing affective stimuli in the environment and individual differences in emotional functioning.

Studies in adult populations point to the anterior cingulate cortex (ACC), which is forms a part of Steinberg's (2004) cognitive-control network and the amygdala and orbitofrontal cortex (OFC), pieces of the socioemotional network, as regions of a neural circuit for emotion-attention interaction. The ACC seems to be particularly involved in shifting attention between emotional and non-emotional stimuli. In one study, greater activation of the ACC was shown when participants attended to the emotional state elicited by an affectively arousing scene than when simply attending to physical aspects of the image (Lane et al., 1997). Yamasaki et al. (2002) also found that the ACC was the only brain region with equivalent responses to attentionally and emotionally manipulated stimuli, emotional scenes in this case. In contrast, amygdala activation seems to be reduced when attention is directed away from an emotional stimulus or higher order cognitive processing is required. Hariri et al. (2000) found that amygdala activation when labeling the emotional content of a face was reduced when compared to a simple matching task of emotional faces that required less cognitive resources. Additionally, Pessoa et al. (2002) found that amygdala activation decreased as attentional demand shifted away from an emotional face stimulus. However, Vuilleumier et al. (2001) provide evidence that amygdala activation to fearful faces is not changed by task motivated attention modulation in contrast to the fusiform gyrus. Finally, the OFC may be the part of the circuit most modulated by *valence* of emotional stimulus under attentional demand. One study (Pessoa et al., 2002) found that OFC activity was modulated by the emotional expression of a face stimulus, which interacted with an attentional demand condition. Thus, the OFC may be most responsible for determining the social significance of the emotional stimulus competing for attentional resources.

Few studies have examined the interaction of emotional and attentional neural networks in a child and adolescent population. Monk et al. (2003) presented adolescent participants with emotional facial expressions of diverse valence but varied attention to the affective content of the face by asking participants to focus on emotional (subjective emotional feeling) vs. non-emotional (nose width) aspects of the face. The authors found that when comparing adolescents and adults, adolescents showed decreased activation of the OFC when attending to subjective emotional feeling, but increased activation in the ACC, OFC, and amygdala when attention was not directed at processing a fearful face. The authors suggest that these results signify adults’ ability to employ relevant brain regions based on attentional demands that may be a developing skill during adolescence. The results of this study suggest that maturation from adolescence to adulthood may involve increased ability to galvanize relevant neural circuits toward goal-directed attention when affectively laden events compete for attention, however this study did not examine maturation from childhood to adolescence, nor did it examine the impact of individual differences in emotional reactivity on these processes. Two additional studies have used a similar paradigm to examine the role of clinical anxiety (McClure et al., 2007) and the related temperamental construct of behavioral inhibition (Perez-Edgar et al., 2007) on emotion–attention interaction, each finding heightened amygdala response in the impaired group when attending to their own subjective fearful feeling. These studies, however, were focused only on the amygdala rather than the emotion-attention circuit as a whole and compared two distinct groups rather than taking a dimensional approach to individual differences.

The goals of the current study were twofold. First, we intended to examine the developing neural circuitry involved in emotion–attention interaction when emotional stimuli were entirely irrelevant to the attentional task, and therefore, only “distractors” in nature. To do this, we developed a novel and child-friendly, socially-oriented, emotion–attention interaction task in which participants were required to count the number of infrequently occurring shape stimuli while also faced with competing, emotionally arousing, but task irrelevant emotional face stimuli in the visual environment. The goal was to create a “social” situation during fMRI scanning with the task of ignoring irrelevant social distractors (i.e. emotional faces) to attend to only basic, non-social, elements of the scene (i.e. shapes). Based on the results of previous research (Pessoa et al., 2002, Monk et al., 2003) indicating that the OFC is particularly sensitive to emotional valence, we hypothesized that OFC activation would be modulated by valence of emotional distractor (i.e. increased activation for emotionally negative stimuli), whereas the ACC and amygdala would show less variation based on emotional distractor stimulus. We also hypothesized that this effect would correlate with age, such that adolescents would be better able to modulate the OFC (i.e. activation to negative stimuli positively correlated with age) in the face of attention grabbing emotional stimuli than children.

The second goal of this study was to assess the effects of individual differences in emotional functioning on emotion–attention interaction. Specifically, emotional reactivity, or variation in the quality and intensity of response to affectively evocative stimuli (Wheeler et al., 1993), may affect engagement of attentional resources when confronted with competing emotional demands. For example, someone who is highly emotionally reactive may have increased subcortical (e.g. amygdala) activation to fearful faces compared to a less emotionally reactive counterpart. In this case, his/her brain may fail to stimulate the relevant cortical circuitry to determine the social significance of the stimulus (e.g. OFC) and to modulate attention away from it (e.g. ACC), resulting in increased behavioral sensitivity to the emotional environment. We hypothesized that individual differences in emotional reactivity in our own sample would correlate with decreased activation of the key areas in the task-identified emotion-attention interaction circuit.

## 2 Materials and methods

All methods for recruitment and participant testing were approved by the institutional review board (IRB) of the University of Pittsburgh.

### 2.1 Participants

Twenty-seven youth who were physically healthy and without personal or family history of mental illness in a first degree relative participated in this study. Participants and their parents completed the Kiddie Schedule for Affective Disorders and Schizophrenia for School-Age Children Depression Rating Scale (KDRS) (Kaufman et al., 1997), the Kiddie Schedule for Affective Disorders and Schizophrenia for School-Age Children Mania Rating Scale (KMRS) (Axelson et al., 2003), and the Screen for Child Anxiety Related to Emotional Disorders (SCARED) (Birmaher et al., 1997) to ensure no current mood/anxiety symptoms. Participants had normal or corrected to normal vision.

Exclusion criteria for participation included neurological disorders, history of head trauma with loss of consciousness, use of medications that may produce CNS effects (e.g. steroids), IQ < 70 (Wechsler Abbreviated Scale of Intelligence) (Weschler, 1999), inability to complete questionnaires in English, pregnancy, claustrophobia, or metal objects in the body. Participants were recruited through local community advertising. Parents/guardians provided written informed consent and youth provided written informed assent prior to study participation. Participants received monetary compensation and a framed picture of their structural neuroimaging scan (Perlman, 2012).

Twenty-three participants provided usable data after data preprocessing (see Section 2.4). Three participants provided only one of two usable runs of data leaving a final 43 usable runs. These participants ranged in age from 8.05 to 16.93 years (*M* = 13.46, SD = 2.86). Participants were 14 male and 9 female and ranged in IQ from 86 to 124 (*M* = 112.78, SD = 9.52).

### 2.2 Emotional reactivity questionnaire

In order to determine each participant's level of emotional reactivity, a parent completed the Children's Affective Lability Scale – CALS (Gerson et al., 1996). The CALS is a 20-item parent report measure developed to assess emotional reactivity in youth aged 6–16. In addition to the overall CALS score, participants are scored on two separate subscales (angry/depressed and disinhibited/impersistent). The CALS has been normed in both typically developing youth and youth presenting with psychiatric disorders.

### 2.3 Paradigm

Participants completed two runs of a 7.1 min, novel, task which was designed to add a social/emotional element to the classic visual/auditory oddball paradigm (Squires et al., 1975). We set up a “social” situation for children in which they were asked to help a peer. Before task participation, children were told the story of a little girl who goes to the grocery store with her mother while carrying a bag of marbles. While at the grocery store, she drops her bag of marbles, which scatter all over the store. The job of the participant was to help the little girl pick up all her marbles. The participant was told to press a button when s/he sees a marble (oddball stimulus), but not to press the button when s/he sees a block (standard stimulus). Additionally, the child was told to keep a mental count of the number of images containing marbles that throughout the duration of the experiment, requiring participants to attend to the basic shapes involved in the task rather than social distractor stimuli. Counting of the oddballs adds an additional cognitive load to the task and minimizes focus on the distractor stimuli.

**Fig. 1:**
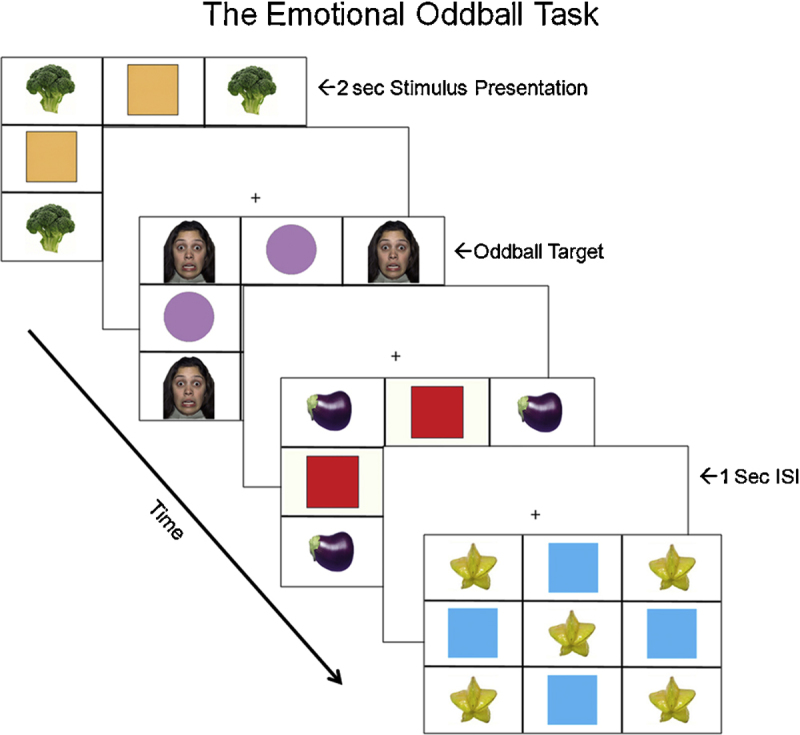
A graphic representation of one fear trial of the Emotional Oddball Task. Participants were asked to press a button every time they saw marbles (oddballs), which occurred approximately every 12 s. Marbles were paired with fearful faces, happy faces, or neutral fruits and vegetables, similar to standard trials.

The task (see Fig. 1) was a slow, event-related design in which each slide stimulus was presented for two seconds with a one second inter-stimulus interval (a black cross hair centered upon a white screen). Each slide stimulus contained a grid in which standard stimuli (blocks) or the infrequently occurring oddball (marbles) were presented in the presence of distractors. Distractors were task irrelevant and designed to briefly provoke a response of the targeted brain circuitry. Standard stimuli (blocks) were always paired with neutral distractors (produce from the grocery store) and oddball stimuli (marbles) were paired with either neutral distractors (produce), positive distractors (people posing happy facial expressions), or negative distractors (people posing fearful facial expressions). Each task run contained 24 oddball trials (8 happy, 8 fearful, 8 neutral) separated by 4–6 standard trials (12–18 s). There were 117 standard trials in each run. Trials were presented in the same order for all subjects. At the end of each run, participants were asked how many marbles they had picked up (i.e. how many slides with marbles they had counted). Three “dummy trials” were also included in which neutral distractors (produce) were paired with standard stimuli (blocks). These trials were included so that subjects did not always associate faces with oddballs. Dummy trials were not included in the analysis.

Face distractor stimuli were chosen from the NimStim Stimulus Set (Tottenham et al., 2009). We chose to use images of produce rather than neutral faces for several reasons. First, there is some evidence that children perceive neutral faces as threatening and have heightened amygdala reactions to these stimuli (Thomas et al., 2001). Second, it was our intention to contrast a social stimulus (i.e. a face) with a non-social stimulus (i.e. produce). Finally, in order to make the emotional distractor truly task-irrelevant, the neutral oddball trials had the same distractor stimuli as standard trials (produce). The use of neutral faces would have indicated to subjects that all oddballs were presented with faces, allowing them to focus on the emotional distractor rather than the basic cognitive task (i.e. marble vs. block).

### 2.4 Neuroimaging data acquisition

Before scanning began, participants practiced the task on a desktop computer and then participated in a mock scanning session to help ensure compliance with the requirement to remain motionless during data collection. This also helped the participants to feel as comfortable as possible while participating in the actual experiment. Participants were trained to remain still while their head motion was projected onto a target inside a replica of our MRI scanner. They were told to “keep their head in the center of the target”. During practice, custom-written software received input from a head motion sensor worn by the participant and used that input to play a sound when the participant moved outside of a set threshold (3 mm; target center). In addition to 10 min of stillness training, participants again practiced the task inside the mock scanner. With the addition of realistic scanner sounds played during the practice session, we were able to closely reproduce the scanning environment in order to familiarize our young participants with fMRI scanning procedures, thereby improving compliance and increasing the likelihood of obtaining high-quality data. The structure of the shortened practice task used on the practice computer and mock scanner was the same, but contained only a few trials that were presented in a different order than the fMRI task.

After mock scanning, neuroimaging data were collected on a 3.0 Tesla Siemens Trio using a 12 channel head coil. Structural 3D axial MPRAGE images were acquired in the same session (TR/TE = 2200/3.29 ms, Flip angle 9°, FOV: 256 mm × 192 mm, Slice thickness: 1 mm, Matrix: 256 × 256, 192 continuous slices). BOLD images were then acquired with a gradient echo EPI sequence for 213 successive brain volumes for each run, covering 39 axial slices (3.2 mm thick, TR/TE = 2000/28 ms/ms, FOV = 205 mm × 205 mm, matrix = 64 × 64; Flip angle 90°. A child-sized, graspable button device recorded participant response.

### 2.5 Data analysis

Data were preprocessed and analyzed using BrainVoyager QX 2.4 (Brain Innovation, Maastricht, The Netherlands). Preprocessing included slice time correction (cubic spline interpolation), alignment of slice (cubic spline interpolation to the first non-discarded scan time), 3-dimensional motion correction (tri-linear interpolation), spatial smoothing (6 mm Gaussian kernel), linear trend removal, and temporal high-pass filtering (fast-Fourier transform based with a cutoff of 3 cycles/time course). The functional data sets were co-registered to the Talairach-transformed (Talairach, 1988) T1-weighted anatomical image series to create a 4-dimensional data representation. Z-transformed participant movement was entered as a covariate of no interest at the individual participant level. None of the 23 participants included in this analysis moved more than 3 mm from their starting head position in any of 6 directions/rotations during the course of data collection.

Our analysis strategy employed a simple, whole-brain, conservative approach. A multi-participant statistical analysis was performed by multiple linear regression of the time course of the BOLD response in each voxel across the whole brain. Regressors were generated to represent the design matrix of the experiment and a general linear model was computed to fit these regressors to each participant's *z*-normalized volume time courses. Model predictors were defined by convolving an ideal boxcar response with a gamma-function model of the hemodynamic response (Friston et al., 1994). Boxcar values were equal to 1 during the oddball events and 0 during the standard events (baseline).

First, we computed a one-way, repeated-measures, ANOVA with 3 levels (valence: Fear, Happy, Neutral) to examine the effect of valence of emotional distractor across the whole brain, relative to standard trials (baseline). Activation maps were visualized on a Talairach-transformed template brain, and displayed at a resolution of 1 mm^3^, with a *p*-value subjected to a multiple-comparison whole-brain correction of FDR(*q*) < 0.05. This procedure deals with the problem of multiple comparisons by automatically identifying a threshold for statistical significance to ensure that, on average, the proportion of false positives among the activated voxels will be less than *q* (Genovese et al., 2002). Next, whole brain post hoc (*t*-test) comparisons were made to compare specific valences of oddball distractor conditions. These tests were subjected to multiple comparison correction of FDR(*q*) < 0.05. Finally, we entered participant age as a covariate of these post hoc comparisons to examine age related changes in emotion–attention interaction.

To examine the impact of emotional reactivity on BOLD signal timecourse, baseline corrected average percent BOLD signal change data were extracted from the functional mask of the effect of emotional distractor valence described above. We examined latency to peak activation by fitting a series of latent growth curve models (LGCM) using Mplus 7.0 (Muthén and Muthén, 2007) using a maximum likelihood estimator. The LGCM provided two latent factors to describe overall level (intercept factor) and rate of change (slope factor) for the extracted percent BOLD signal change. These factors were then regressed on emotional reactivity scores (CALS). Model fit was evaluated using the *χ*^*2*^ goodness of fit test, comparative fit index (CFI), Tucker–Lewis index (TLI), and root-mean-square error of approximation (RMSEA). For CFI and TLI, we used the conventional cutoff ≥90 for acceptable fit, and ≥95 for good fit. RMSEA values between .05 and .08 represent acceptable fit, while values <05 indicate good fit (McDonald and Ho, 2002).

Data were inspected for outliers by examining standardized residuals resulting from regressions of the emotional reactivity score predicting each BOLD signal change measurement (4–7 s post-stimulus presentation). Scatterplots of residuals versus predicted values were also visually inspected to identify outliers. Standardized residuals were no greater than | 3 |, suggesting no extreme cases. Visual inspection of scatterplots of residuals versus predicted values also did not reveal any extreme cases.

## 3 Results

### 3.1 Emotional reactivity

Parent report of emotional reactivity based on the Child Affect Lability Scale-CALS ranged from 0–6 (mean = 1.22, SD = 1.783) out of a possible score of 80. Low scores for a typically developing group (*n* = 212, mean = 8.94, SD = 9.02) were also reported in the original psychometric evaluation of the measure (Gerson et al., 1996). CALS score did not correlate with age [*r*(25) = .14, *p* = .50] nor did it correlate with IQ [*r*(25) = .22, *p* = .27] in our sample. CALS score also did not differ between male and female participants [*t*(25) = −1.206, *p* = .239]. On individual CALS subscales, participants scored mean = .86, SD = 1.25 for the angry/depressed subscale and mean = .82, SD = 1.14 for the disinhibited/impersistent subscale.

### 3.2 Task performance

Oddball detection accuracy and reaction time were recorded for all participants and averaged across usable runs. One participant did not provide accuracy or reaction time data due to a technical error. Accuracy rates for detecting the oddball stimulus in the 22 remaining participants did not differ significantly for the three conditions: 86% (happy); 89% (fear); 88% (neutral); [*F*(2,42) = 1.83, *p* = .17]. There was, however, a significant difference in reaction time [*F*(2,42) = 8.38, *p* = .001] with participants responding more quickly to happy (636.70 ms) and fear (631.04 ms) oddballs compared to neutral oddballs (680.05 ms). This was possibly due to the novelty or emotional arousal produced by the face distractor that did not appear on standard trials. Average oddball detection accuracy correlated with age [*r*(20) = .51, *p* = .02], but did not correlate with IQ. Older participants were more accurate than younger participants in oddball detection. Average reaction time did not correlate with age or IQ. Participants reported counting an average of 21.74 marbles/oddballs (correct answer 24; range 8–25), which did not correlate with age or IQ. The number of marbles counted did correlate positively with accuracy [*r*(20) = .61, *p* = .002] and negatively with reaction time [*r*(20) = −.54, *p* = .01], but did not correlate with BOLD measures in any region of interest.

### 3.3 Neural activity

**Fig. 2:**
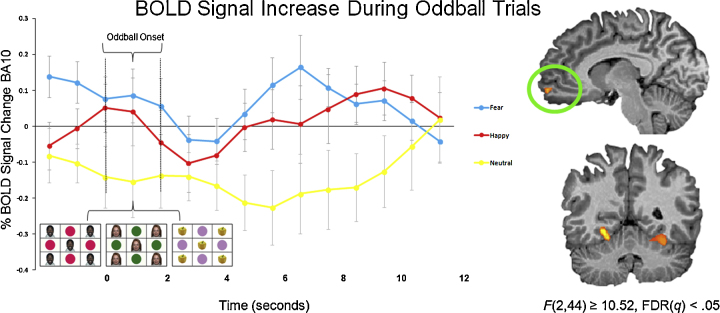
Results of a whole-brain, one-way ANOVA revealed an effect of emotional distractor condition in the left OFC (BA 10) and bilateral parahippocampal gyrus (Right Panel). Extracted, baseline corrected, percent BOLD signal change timecourse in the OFC revealed a peak in activation for the fearful condition at approximately 7 s post stimulus onset (Left Panel).

The one-way, 3-level (valence: Happy, Fear, Neutral) ANOVA revealed an overall effect in four regions of the brain with a minimum cluster size of 100 1 mm^3^ voxels [*F*(2,44) ≥ 10.52, *q* < .05; Fig. 2 (right panel)]. Regions were localized to the right parahippocampal gyrus, left fusiform gyrus, right fusiform gyrus, and left medial prefrontal cortex (see Table 1 for statistical information on individual clusters). Extracted percent BOLD signal change in the medial prefrontal/orbitofrontal cortex, the only region in the hypothesized emotion–attention interaction circuit displaying an effect, indicated significant peak activation for the fearful oddball condition in contrast to the neutral oddball condition (Fig. 2, left panel). Results of a bilateral amygdala region of interest analysis did not find a main effect of condition at the *p* < .05, uncorrected level.

**Table 1:** Neural activity significant cluster information.^a^

	Region	BA	Peak voxel	Size (1mm^3^)	Average stat value
Main effect emotional valence:	Right PHG	19	*x* = 23, *y* = −44, *z* = −9	683	*F* = 13.8, *p* < .0001
	Left FG	19	*x* = −31, *y* = −59, *z* = −12	6178	*F* = 13.9, *p* < .0001
	Right FG	19	*x* = 23, *y* = −59, *z* = −12	182	*F* = 12.1, *p* < .0001
	Left mPFC	10	*x* = −7, *y* = 61, *z* = −9	142	*F* = 11.8, *p* = .0001
Fear vs. neutral contrast:	Right PHG	19	*x* = 20, *y* = −44, *z* = −12	750	*F* = −4.7, *p* < .0001
	Left FG	19	*x* = −31, *y* = −56, *z* = −12	7433	*F* = −4.6, *p* < .0001
	Right FG	19	*x* = 23, *y* = −59, *z* = −12	254	*F* = −4.4, *p* < .0001
	Left mPFC	10	*x* = −7, *y* = 61, *z* = −9	248	*F* = 4.4, *p* = .0001
Happy vs. neutral contrast:	Right PHG	19	*x* = 23, *y* = −44, *z* = −9	245	*F* = −4.7, *p* < .0001
	Left PHG	19	*x* = −31, *y* = −59, *z* = −12	1514	*F* = −4.8, *p* < .0001
	Left OG	19	*x* = −34, *y* = −86, *z* = 0	769	*F* = −4.7, *p* < .0001
Fear vs. happy contrast:	–	–	–	–	–

^a^ Whole map statistical threshold set at FDR(*q)* < .05, 100 1 mm^3^ voxel extent. PHG: parahippocampal gyrus, FG: fusiform gyrus, mPFC: medial prefrontal cortex, OG: occipital gyrus.

**Fig. 3:**
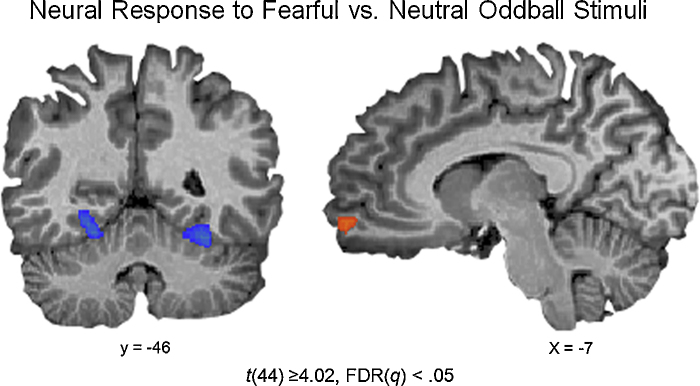
A whole-brain contrast of fearful vs. neutral oddball trials revealed increased activation in the OFC (BA 10) for fearful distractors (red) and increased activation in the bilateral parahippocampal gyrus for neutral distractors (blue). (For interpretation of the references to color in this figure legend, the reader is referred to the web version of the article.)

Results of a whole-brain fear vs. neutral contrast revealed four regions of significant difference that were larger than 100 1 mm^3^ voxels [*t*(44) ≥ 4.02, *q* < .05; Fig. 3]. The right parahippocampal gyrus and left and right fusiform gyrus were significantly more active for neutral oddballs, while the left medial prefrontal cortex was more active for fearful oddballs over neutral oddballs. For the whole-brain happy vs. neutral contrast, results were similar to the fear > neutral contrast except that there was no effect found in any region of the hypothesized emotion–attention interaction circuit. The right and left parahippocampal gyrus and the left middle occipital gyrus were significantly more active for neutral oddballs [*t*(44) ≥ 4.35, *q* < .05]. For the fear vs. happy contrast, no significant clusters of activation were found at the FDR(*q*) < .05 threshold, nor were they found at the uncorrected *p* < .001 threshold.

Results of a whole-brain ANCOVA analysis with participant age as a covariate revealed a cluster (1004 1 mm^3^ voxels) in which the difference between fear and neutral activation correlated positively with age [*r*(21) = .61, *p* = .002, uncorrected]. This area was in the approximate OFC location of our overall effect and the effect of our fear vs. neutral contrast (BA 11, peak voxel: *x* = 8, *x* = 46, *x* = −18). Note, however, that the whole-brain map was set at an uncorrected *p* < .05 threshold.

### 3.4 Emotional reactivity and within-individual increases in BOLD signal in fearful condition

To evaluate the impact of emotional reactivity on BOLD signal rate of increase for the fearful oddball condition, the within-individual trajectories of the baseline corrected, extracted percent BOLD signal change in the OFC region, found earlier to be modulated by emotional distractor valence (see Fig. 2), were examined. A linear model of growth covering the post-stimulus presentation period spanning the lowest point of the BOLD signal change to the peak BOLD signal change (i.e. 4 s to 7 s post-stimulus presentation onset) provided an excellent fit to the data (*χ*^2^_(4)_ = 1.42, *p* = .841; RMSEA < .001; CFI = 1.00; TLI = 1.00). Percent BOLD signal change in this region of the OFC, from 4 to 7 s post-stimulus presentation, increased significantly (*M*_*s*_ = .07, SE = .02, *p* = .002), indicating a steady increase in percent BOLD signal change during this time period. The *M*_*s*_ value of 0.07 can be interpreted as the increase in percent BOLD signal change per second (0.07 × 3 [T7–T4 = 3 s] = .21), which corresponds to a 21% increase in percent BOLD signal change across 4–7 s post-stimulus presentation. The variances for the intercept and slope were *D*_*i*_ = .14, *SE* = 04, *p* = .001, and *D*_*s*_ = .01, SE = .01, *p* < .001, respectively, indicating substantial variation across children in initial percent BOLD signal change and percent BOLD signal change trajectory.

**Fig. 4:**
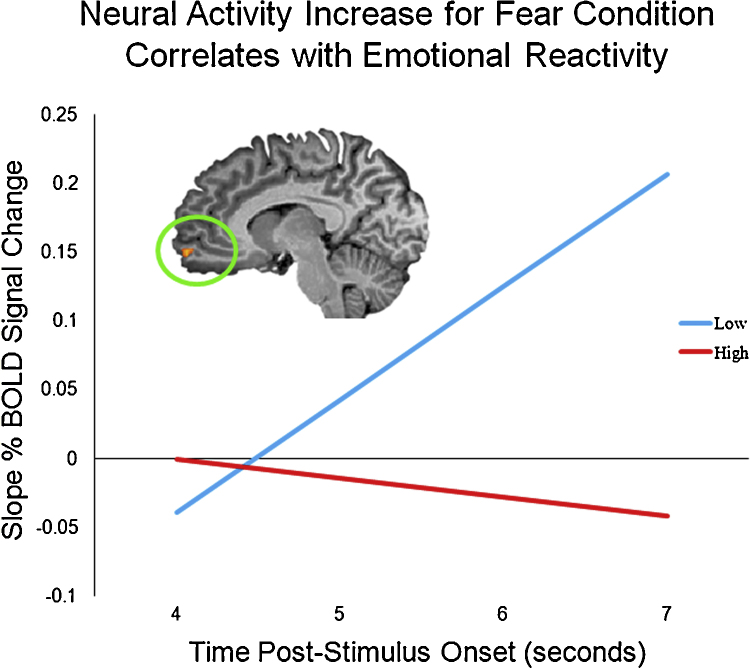
Estimated growth curves of high and low levels of emotional reactivity on the BOLD signal rise to peak activation for the fear condition. For illustration purposes, the High Emotional Reactivity group represents the upper quartile of parent report scores. Low emotional reactivity predicted a steeper slope to hemodynamic response peak in the fear condition, suggesting that individual difference in emotional reactivity moderate the use of the OFC to modulate attention in the presence of emotional distractors. Note that data is interpolated to 1 s intervals for the purposes of display.

Next, emotional reactivity scores were entered as a predictor of the intercept and slope factors of the LGCM. The conditional model also provided excellent fit to the data (*χ*^2^_(6)_ = 4.81, *p* = .568; RMSEA < .001; CFI = 1.00; TLI = 1.00). Emotional reactivity significantly predicted the slope factor (*β* = −.24, SE = .10 *p* = .016), suggesting that children with higher levels of emotional reactivity had a slower rate of increase to peak activation for the fearful oddball condition (Fig. 4). Emotional reactivity did not significantly predict the intercept factor (*β* = .14, SE = .14 *p* = .429), suggesting that emotional reactivity does not impact overall level of activation during this period. The subscales of emotional reactivity, angry/depressed and disinhibited/impersistent, were not significantly related to the intercept or slope factors.

## 4 Discussion

Our investigation employed a novel, child-oriented, social task to study the neural circuitry involved in emotion–attention interaction during childhood and adolescence. We found that activation in the OFC was modulated by the emotional valence of task irrelevant stimuli. We also found that BOLD signal increase trajectory in this region, during fearful distractors, correlated with parent reported emotional reactivity.

Our first hypothesis was that OFC activation would be modulated by the valence of emotional distractor (i.e. increased activation for negative stimuli), whereas the ACC and amygdala would show less variation based on valence of emotional distractor stimulus. Unlike the majority of previous neuroimaging studies investigating emotion–attention interaction (Vuilleumier et al., 2001, Pessoa et al., 2002, Yamasaki et al., 2002, Monk et al., 2003), we chose a task-irrelevant distractor that varied in emotional valence (fearful, happy, neutral). Our conservative, whole-brain analyses found that the OFC was the only region in the hypothesized attention–emotion interaction circuit that was responsive to emotional valence of the distractor (increased activation for negative stimuli), in addition to the fusiform gyrus (i.e. fusiform face area (Kanwisher et al., 1997)) and parahippocampal gyrus (i.e. parahippocampal place area (Epstein and Kanwisher, 1998)) which likely selectively responded to faces and objects respectively. One must note, however, that the lack of differentiation of the OFC for happy vs. fearful stimuli may point to the role of the OFC in dissociating social vs. non-social stimuli. These results support Steinberg's model of adolescent social decision making (Steinberg, 2004) by involving the OFC, a critical region in the socioemotional circuit in the process of focusing attention away from emotional stimuli. Results in the OFC replicate those of previous studies in adults (Pessoa et al., 2002) and adolescents (Monk et al., 2003) finding increased OFC activation for negative emotional stimuli.

Further, we hypothesized that this effect would correlate with age, such that adolescents would be more able to modulate OFC in the face of attention grabbing negative stimuli than children. We found some, albeit uncorrected, evidence that difference in processing of fearful and neutral distractors in the OFC region increased with age, a finding that replicates and extends the findings of Monk and colleagues (Monk et al., 2003), who established that adolescents were “immature” in the use of this region compared to adults. These findings also support the work of Casey and Durston (2006) (Casey and Durston, 2006, Durston and Casey, 2006) and Ernst and Fudge (2009) who describe the medial prefrontal cortex/OFC as a modulation region of subcortical areas (e.g. amygdala), which becomes more specialized during adolescent development. If this finding replicates to larger and more impaired clinical populations (e.g. finding decreased use of this region across age or lack of connectivity to other regions in the emotion-attention circuit), results may imply dysfunction between the cognitive control-and socioemotional circuits in those who develop social problems (i.e. juvenile delinquency) and various psychiatric disorders during adolescence.

A second focus of our study was on individual differences in emotional reactivity and its effect on emotion–attention interaction circuitry. Although behavioral studies have found links between attention modulation toward and away from emotionally evocative stimuli and various personality traits (Mogg and Bradley, 1999, Bradley et al., 2000, Fox et al., 2001, Yiend and Mathews, 2001, Isaacowitz, 2005, Lindstrom et al., 2009, Perlman et al., 2009), the neural substrates of these processes remain relatively unknown (Vuilleumier, 2005). This is especially true of childhood and adolescent samples in which personality development and related neural underpinnings may still be maturing. Some studies, employing attention modulation tasks in which adolescent subjects must divert their attention between emotional faces presented simultaneously, have found increased activation in the ventrolateral PFC/OFC related to trait anxiety (Telzer et al., 2008) and anxiety disorders (Monk et al., 2006) during orientation away from angry faces. These studies imply the role of the OFC region in modulating emotional information that is personally relevant, a finding replicated in our own study. We found that, for fearful emotional distractors, parent reported emotional reactivity correlated with the trajectory of peak BOLD signal activation after stimulus onset, suggesting that participants with lower levels of emotional reactivity had increased rate of BOLD signal activation in this area. This could imply that the increase in adaptive use of this region is not only associated with age (Monk et al., 2003), but is also tightly coupled with adaptive emotional functioning. Examined within the context of previous work, these results could imply a neural mechanism for the often observed individual differences in attentional orientation toward and away from emotional stimuli (Isaacowitz, 2005, Lindstrom et al., 2009, Perlman et al., 2009), even in the absence of psychopathology.

Although our results examined individual differences within a normative child and adolescent sample, emotional reactivity is a transdiagnostic construct that is relevant for understanding the development and maintenance of a variety of mood, anxiety, and personality disorders, including depression, bipolar disorders, post-traumatic stress disorder, and borderline personality disorder (Koenigsberg, 2010). Individuals with high levels of emotional reactivity are likely to have both over- and under-responsive emotional feedback systems that may interfere with attention–emotion interaction (Hamann and Canli, 2004). Pertaining specifically to development, even normative individual differences in emotional reactivity during pubertal transitions may precipitate the emergence of psychopathology among vulnerable adolescents, which could contribute to the increases in affective disturbances and psychological disorders during this period (Spear, 2000). We found neural markers of individual differences in emotional reactivity within a normative sample, which, if applied to samples reporting sub-optimal emotional reactivity, may elucidate developmental trajectories toward early-onset psychopathology and even, potentially, contribute to the development of therapeutic interventions in these populations.

Our results are promising; however, they are not without limitations. First, we intentionally included only one level of attention modulation (task-irrelevant distractors) rather than varying attention toward and away from an emotional stimulus (Yamasaki et al., 2002, Monk et al., 2003). This strategy proved to be particularly efficient in provoking the modulation of the OFC due to emotional valence of the distractor, but was unsuccessful at modulating the amygdala and ACC. Even an uncorrected region of interest analysis in the amygdala failed to show a main effect of emotional distractor, which is likely due to a lack of attention modulation condition (Pessoa et al., 2002). Future use of this paradigm may employ an attention modulation condition to better examine all areas of the attention–emotion interaction circuit and paint a more complete picture of how neural connectivity amongst these regions relates to individual differences in emotional reactivity. Further, aside from unanalyzed dummy trials, faces were never paired with standard stimuli, which could indicate that the OFC is particularly sensitive to a social stimulus rather than the emotional content present in stimuli. Second, although designed to investigate one aspect of visual attention, our study did not measure visual behavioral data (e.g. eye-tracking). It is therefore, impossible to determine the level of visual attention to emotional distractors, especially in correlation with emotional reactivity, even though these stimuli may be task irrelevant. Visual attention to emotional stimuli is known to correlate with other measures of emotional reactivity (Perlman et al., 2009) and should be considered in future studies. Finally, our sample size was small, recruited to eliminate psychiatric disorders, and on the low end of the emotional reactivity spectrum. Although emotional reactivity score for our participants was comparable to what has been reported in typically developing samples (Gerson et al., 1996), our sample was particularly affectively resilient (i.e. extremely low emotional reactivity scores), which serves to emphasize the sensitivity of the mechanisms examined, but also raises questions regarding replication of results in other samples. Future studies may investigate larger samples chosen to represent a wider range of emotional reactivity, both in the context of normative individual differences and those of psychiatric disorders. Studies are underway to employ this novel task in longitudinal child and adult samples to investigate emotion–attention interaction in the context of personality variation, risk for psychopathology, and clinical diagnosis.

## Conflict of interest statement

The authors declare no conflicts of interest.

## Appendix A

*Author's Note*: Longitudinal Assessment of Manic Symptoms (LAMS) Consortium Members: Jorge R. C. Almeida, L. Eugene Arnold, David Axelson, Genna Bebko, Michele A. Bertocci, Boris Birmaher, Lisa Bonar, Christine Demeter, Vaibhav A. Diwadkar, Jay C. Fournier, Robert L. Findling, Mary. A Fristad, Mary Kay Gill, Amanda K. Hinze, Scott K Holland, Sarah M. Horwitz, Robert. A. Kowatch, Susan B. Perlman, Mary L. Phillips, Claudiu Schirda, Jeffrey L. Sunshine, Michael Travis, Amelia Versace, Eric A. Youngstrom

## References

[bib0005] Axelson D., Birmaher B.J. (2003). A preliminary study of the Kiddie Schedule for Affective Disorders and Schizophrenia for School-Age Children mania rating scale for children and adolescents. J. Child Adolesc. Psychopharmacol..

[bib0010] Birmaher B., Khetarpal S. (1997). The Screen for Child Anxiety Related Emotional Disorders (SCARED): scale construction and psychometric characteristics. J. Am. Acad. Child Adolesc. Psychiatry.

[bib0015] Bradley B.P., Mogg K. (2000). Covert and overt orienting of attention to emotional faces in anxiety. Cogn. Emot..

[bib0020] Casey B.J., Castellanos F.X. (1997). Implication of right frontostriatal circuitry in response inhibition and attention-deficit/hyperactivity disorder. J. Am. Acad. Child Adolesc. Psychiatry.

[bib0025] Casey B.J., Durston S. (2006). From behavior to cognition to the brain and back: what have we learned from functional imaging studies of attention deficit hyperactivity disorder?. Am. J. Psychiatry.

[bib0030] Durston S., Casey B.J. (2006). What have we learned about cognitive development from neuroimaging?. Neuropsychologia.

[bib0035] Eigsti I.M., Zayas V. (2006). Predicting cognitive control from preschool to late adolescence and young adulthood. Psychol. Sci..

[bib0040] Elliott R. (2003). Executive functions and their disorders. Br. Med. Bull..

[bib0045] Ellis L.K., Rothbart M.K. (2004). Individual differences in executive attention predict self-regulation and adolescent psychosocial behaviors. Ann. N. Y. Acad. Sci..

[bib0050] Epstein R., Kanwisher N. (1998). A cortical representation of the local visual environment. Nature.

[bib0055] Ernst M., Fudge J.L. (2009). A developmental neurobiological model of motivated behavior: anatomy, connectivity and ontogeny of the triadic nodes. Neurosci. Biobehav. Rev..

[bib0060] Fox E., Russo R. (2001). Do threatening stimuli draw or hold visual attention in subclinical anxiety?. J. Exp. Psychol.: Gen..

[bib0065] Friston K.J., Holmes A.P. (1994). Statistical parametric maps in functional imaging: a general linear approach. Hum. Brain Mapp..

[bib0070] Genovese C.R., Lazar N.A. (2002). Thresholding of statistical maps in functional neuroimaging using the false discovery rate. Neuroimage.

[bib0075] Gerson A.C., Gerring J.P. (1996). The Children's Affective Lability Scale: a psychometric evaluation of reliability. Psychiatry Res..

[bib0080] Giedd J.N., Blumenthal J. (1999). Brain development during childhood and adolescence: a longitudinal MRI study. Nat. Neurosci..

[bib0085] Goldman-Rakic P.S. (1987). Development of cortical circuitry and cognitive function. Child Dev..

[bib0090] Hamann S., Canli T. (2004). Individual differences in emotion processing. Curr. Opin. Neurobiol..

[bib0095] Hare T.A., Tottenham N. (2008). Biological substrates of emotional reactivity and regulation in adolescence during an emotional go-nogo task. Biol. Psychiatry.

[bib0100] Hariri A.R., Bookheimer S.Y. (2000). Modulating emotional responses: effects of a neocortical network on the limbic system. Neuroreport.

[bib0105] Isaacowitz D.M. (2005). The gaze of the optimist. Pers. Soc. Psychol. Bull..

[bib0110] Kanwisher N., McDermott J. (1997). The fusiform face area: a module in human extrastriate cortex specialized for face perception. J. Neurosci..

[bib0115] Kaufman J., Birmaher B. (1997). Schedule for Affective Disorders and Schizophrenia for School-Age Children-Present and Lifetime Version (K-SADS-PL): initial reliability and validity data. J. Am. Acad. Child Adolesc. Psychiatry.

[bib0120] Koenigsberg H.W. (2010). Affective instability: toward an integration of neuroscience and psychological perspectives. J. Pers. Disord..

[bib0125] Lane R.D., Fink G.R. (1997). Neural activation during selective attention to subjective emotional responses. Neuroreport.

[bib0130] Lindstrom K.M., Guyer A.E. (2009). Normative data on development of neural and behavioral mechanisms underlying attention orienting toward social-emotional stimuli: an exploratory study. Brain Res..

[bib0135] Luna B. (2009). Developmental changes in cognitive control through adolescence. Adv. Child Dev. Behav..

[bib0140] McClure E.B., Monk C.S. (2007). Abnormal attention modulation of fear circuit function in pediatric generalized anxiety disorder. Arch. Gen. Psychiatry.

[bib0145] McDonald R.P., Ho M.-H.R. (2002). Principles and practice in reporting structural equation analyses. Psychol. Methods.

[bib0150] Mogg K., Bradley B.P. (1999). Orienting of attention to threatening facial expressions presented under conditions of restricted awareness. Cogn. Emot..

[bib0155] Monk C.S., McClure E.B. (2003). Adolescent immaturity in attention-related brain engagement to emotional facial expressions. Neuroimage.

[bib0160] Monk C.S., Nelson E.E. (2006). Ventrolateral prefrontal cortex activation and attentional bias in response to angry faces in adolescents with generalized anxiety disorder. Am. J. Psychiatry.

[bib0165] Muthén L., Muthén B. (2007). Mplus. User's Guide.

[bib0170] Perez-Edgar K., Roberson-Nay R. (2007). Attention alters neural responses to evocative faces in behaviorally inhibited adolescents. Neuroimage.

[bib0175] Perlman S.B. (2012). Neuroimaging in child clinical populations: considerations for a successful research program. J. Am. Acad. Child Adolesc. Psychiatry.

[bib0180] Perlman S.B., Morris J.P. (2009). Individual differences in personality predict how people look at faces. PLoS ONE.

[bib0185] Pessoa L., McKenna M. (2002). Neural processing of emotional faces requires attention. Proc. Natl. Acad. Sci. U. S. A..

[bib0190] Rubia K., Smith A.B. (2003). Right inferior prefrontal cortex mediates response inhibition while mesial prefrontal cortex is responsible for error detection. Neuroimage.

[bib0195] Spear L.P. (2000). The adolescent brain and age-related behavioral manifestations. Neurosci. Biobehav. Rev..

[bib0200] Squires N.K., Squires K.C. (1975). Two varieties of long-latency positive waves evoked by unpredictable auditory stimuli in man. Electroencephalogr. Clin. Neurophysiol..

[bib0205] Steinberg L. (2004). Risk taking in adolescence – What changes, and why?. Ann. NY Acad. Sci..

[bib0210] Steinberg L. (2007). Risk taking in adolescence – New perspectives from brain and behavioral science. Curr. Dir. Psychol. Sci..

[bib0215] Talairach J.T.P. (1988). Co-Planar Stereotaxic Atlas of the Human Brain: 3 Dimensional Proportional System: An Approach to Cerebral Imaging.

[bib0220] Telzer E.H., Mogg K. (2008). Relationship between trait anxiety, prefrontal cortex, and attention bias to angry faces in children and adolescents. Biol. Psychol..

[bib0225] Thomas K.M., Drevets W.C. (2001). Amygdala response to facial expressions in children and adults. Biol. Psychiatry.

[bib0230] Tottenham N., Tanaka J.W. (2009). The NimStim set of facial expressions: judgments from untrained research participants. Psychiatry Res..

[bib0235] Vuilleumier P. (2005). How brains beware: neural mechanisms of emotional attention. Trends Cogn. Sci..

[bib0240] Vuilleumier P., Armony J.L. (2001). Effects of attention and emotion on face processing in the human brain: an event-related fMRI study. Neuron.

[bib0245] Weschler D. (1999). Weschler Abbreviated Scale of Intelligence (WASI).

[bib0250] Wheeler R.E., Davidson R.J. (1993). Frontal brain asymmetry and emotional reactivity: a biological substrate of affective style. Psychophysiology.

[bib0255] Yamasaki H., LaBar K.S. (2002). Dissociable prefrontal brain systems for attention and emotion. Proc. Natl. Acad. Sci. U. S. A..

[bib0260] Yiend J., Mathews A. (2001). Anxiety and attention to threatening pictures. Q. J. Exp. Psychol. A.

